# Fetal Functional Brain Age Assessed from Universal Developmental Indices Obtained from Neuro-Vegetative Activity Patterns

**DOI:** 10.1371/journal.pone.0074431

**Published:** 2013-09-18

**Authors:** Dirk Hoyer, Florian Tetschke, Susan Jaekel, Samuel Nowack, Otto W. Witte, Ekkehard Schleußner, Uwe Schneider

**Affiliations:** 1 Jena University Hospital, Biomagnetic Center, Hans Berger Department of Neurology, Jena, Germany; 2 Jena University Hospital, Department of Obstetrics, Jena, Germany; 3 Jena University Hospital, Integrated Research and Treatment Center, Center for Sepsis Control and Care (CSCC), Jena, Germany; University of Adelaide, Australia

## Abstract

Fetal brain development involves the development of the neuro-vegetative (autonomic) control that is mediated by the autonomic nervous system (ANS). Disturbances of the fetal brain development have implications for diseases in later postnatal life. In that context, the fetal functional brain age can be altered. Universal principles of developmental biology applied to patterns of autonomic control may allow a functional age assessment. The work aims at the development of a fetal autonomic brain age score (fABAS) based on heart rate patterns. We analysed n = 113 recordings in quiet sleep, n = 286 in active sleep, and n = 29 in active awakeness from normals. We estimated fABAS from magnetocardiographic recordings (21.4–40.3 weeks of gestation) preclassified in quiet sleep (n = 113, 63 females) and active sleep (n = 286, 145 females) state by cross-validated multivariate linear regression models in a cross-sectional study. According to universal system developmental principles, we included indices that address increasing fluctuation range, increasing complexity, and pattern formation (skewness, power spectral ratio VLF/LF, pNN5). The resulting models constituted fABAS. fABAS explained 66/63% (coefficient of determination R^2^ of training and validation set) of the variance by age in quiet, while 51/50% in active sleep. By means of a logistic regression model using fluctuation range and fetal age, quiet and active sleep were automatically reclassified (94.3/93.1% correct classifications). We did not find relevant gender differences. We conclude that functional brain age can be assessed based on universal developmental indices obtained from autonomic control patterns. fABAS reflect normal complex functional brain maturation. The presented normative data are supplemented by an explorative study of 19 fetuses compromised by intrauterine growth restriction. We observed a shift in the state distribution towards active awakeness. The lower WGA dependent fABAS values found in active sleep may reflect alterations in the universal developmental indices, namely fluctuation amplitude, complexity, and pattern formation that constitute fABAS.

## Introduction

Since fetal brain development has consequences for the entire life, prenatal developmental disturbances need to be identified and understood at an early stage. In that regard, the fetal neuro-vegetative (autonomic) control plays an important role. According to the concept of “fetal programming”, fetal stress, among other factors, can permanently change the fetal brain development and cause diseases in later life, e.g. [1], [2]. A corresponding reprogramming of the stress system with its branches hypothalamic-pituitary-adrenal (HPA) axis and autonomic nervous system (ANS) also influences the related heart rate variability (HRV) patterns. Since heart rate is one of the few signals that can be obtained non-invasively from the fetus, HRV analysis is uniquely suited to assess the fetal functional brain development. So far, even the normal fetal development of autonomic control cannot be assessed precisely.

Traditionally, different frequencies of heart rate fluctuations were assigned to vagal and sympathetic activity rhythms [3]. Their interrelationships are approximated by their ratio as index of sympatho-vagal balance, and more comprehensively by complexity indices that may reflect their complex and nonlinear interrelationships, e.g. [4], [5]. The analysis of the time scales of the heart rate fluctuations provided further insights into the complex functional organization of neuro-vegetative (autonomic) control [6]–[10].

Those approaches also apply in the evolving and developing fetal autonomic control in the second half of gestation. Increasing fluctuation amplitudes, increasing as well as decreasing complexity, and the differentiation of behavioral pattern were reported to be dependent on gestational age [11]–[13]. There is an indication that those results can be interpreted according to universal rules of developmental biology [14].

Universal principles of complex system behaviour provide a link between self-organization and adaptation, phylogeny, ontogeny, and individual development [15]–[19]. HRV as marker of ANS activity can be considered as order parameter [20] of the system “fetal organism/autonomic control”. Predominant universal principles of development are (i) increasing fluctuation amplitude, (ii) increasing complexity, and (iii) pattern formation. Corresponding HRV indices may reflect the maturation of the fetal autonomic control system. Since the emergence and forming of fetal behavioural states is part of the maturation, we investigated those characteristics in the most frequently appearing states that correspond to quiet and active sleep according to previous analyses [13], [14], [21]–[24]. A joint analysis of those characteristics with regard to the development of fetal autonomic control is pending.

The present work aims at the design of a functional fetal autonomic brain age score (fABAS) based on multivariate analyses of universal developmental indices calculated from fetal heart rate patterns.

Intrauterine growth restriction (IUGR) represents a condition that, in the context of neurovegetative development, may serve as a model of chronic lack of nutritional supply that may be well enough characterized by clinical means based on ultrasound investigation [25]. IUGR may be caused by a variety of causes – in the sense considered here it is defined as the clinical expression of the fetus not exploiting its genetic potential of growth and development due to chronic placental insufficiency. IUGR is associated with an increased perinatal morbidity and mortality and the optimized timing of delivery so far remains the only therapeutic option once the condition has manifested itself [25]–[27]. First reports of changed univariate HRV characteristics indicate altered autonomic control in association with IUGR. However, the results are heterogeneous due to different data length and fetal behavioural states investigated [28]–[30]. In order to explore the potentials of fABAS in augmenting the assessment of potentially impaired neurovegetative development [31], the normative data presented here are supplemented by data from a cohort of fetuses who suffered from IUGR.

## Methods

### Subjects and Data Acquisition

The study was approved by the Local Ethics Committee of the Friedrich Schiller University. All subjects gave their written consent to perform the study.

In a cross-sectional prospective observational study 428 normal singleton fetuses, healthy according to standard obstetric observation methods in nonstress situation, obtained in the Biomagnetic Center (Jena University Hospital), were allocated. In all these cases standard maternity documents were revised at the time of investigation to confirm gestational age (weeks of gestational age (WGA) according to last menstrual period verified by first trimester fetal crown-rump-length) and the normal course of the ongoing pregnancy. The following conditions served as exclusion criteria to the normal cohort: Maternal: known heart diseases, diabetes mellitus of the mother, maternal medication affecting cardiac function/rhythm, abuse of nicotine, alcohol or drugs, previous administration of synthetic glucocorticoids, uterine contractions during the recording. Fetal: known chromosomal abnormalities, sonographically identified malformations, fetal cardiac arrhythmias.

Perinatal outcome data were available in around 65% of the studied cases (Table 1):

**Table 1:** Neonatal outcome characteristics, mean (standard deviation), [min-max].

	Normal	IUGR
age of birth (WGA)	39.6 (1.6), [31–42]	35.6 (3.3), [26.4–39.6]
weight of birth (g)	3438 (503), [1460–4700]	1945 (703), [480–3220]
APGAR score after 5 min	9.1 (1.1), [5]–[10]	8.1 (1.6), [5]–[10]
ph value of umbilical cord blood	7.25 (0.08), [6.97–7.47]	7.30 (0.06), [7.13–7.37]

There was one case of preterm delivery at 32+4 WGA (birth weight 1460 g). This fetus was studied at 31.0, at this time there were no signs of threatened preterm labour.

In one neonate (40 WGA, birth weight 3070 g, APGAR at 5 min 8) at birth an acidotic pH value (6.97) was observed in arterial cord blood.

IUGR was assumed when a sonographically estimated fetal weight was observed below the 10^th^ percentile in combination with pathologic uteroplacental perfusion on Doppler ultrasound beyond 24 WGA (mean pulsatility index in the Aa. uterinae >1.5 and/or bilateral notching) [25], [32]. N = 19 cases were recruited consecutively on the occasion; the timing of investigation serving as a surrogate marker for onset and severity of the condition. Data were then drawn from the database for this preliminary explorative data analysis presented here.

Outcome parameters were available in all 19 cases showing that one case was wrongly classified as IUGR (birth weight 3220 g at 39+4 WGA), which was due to underestimated fetal weight on ultrasound at the time of magnetocardiographic investigation (38+1 WGA). The case was not removed from the studied sample.

### Study Protocol and Standard Operation Procedure

All recordings were obtained using a standard procedure designed in a prospective study in 2006 (DFG, HO 1634 12-2, Schn 775/2–3). The resulting study data base that includes recording, maternal and fetal characteristics and neonatal outcome was built up until 2013.

All magnetocardiographic recordings were performed during daytime over a period of 30 min. The pregnant women were positioned supine or with a slight twist to either side to prevent compression of the inferior vena cava by the pregnant uterus. The Dewar containing the magnetometers was positioned with its curvature above the fetal heart after sonographic localization as close to the maternal abdominal wall as possible without direct contact.

In the recordings the heart beats were detected and normal-to-normal (NN) beat intervals series calculated. The NN series were screened for artifacts, arrhythmias and non-stationarities and 10 min intervals of active (according to 2F) and quiet (according to 1F) sleep and active awakeness (according to 4F) were selected after a consensus decision by three independent obstetricians blinded to heart rate analysis according to an advanced version of standard criteria [14], [21], [24] that was extended as follows. Since some data sets of active states for gestational age <32 weeks showed fluctuation ranges more likely according to 4F than 2F, we extended the discrimination of 2F and 4F equivalents to the entire investigated age range of 21.4–40.7 WGA.

### State Classification Criteria

#### Quiet state/HRP I (interpreted as quiet sleep 1F)

Stable fetal heart rate (fHR) (variation of visually determined floating baseline <10 bpm/3 min) with a small oscillation bandwidth (< ±5 bpm from floating baseline fHR), isolated (maximum 2 per 10 min) accelerations (>15 bpm over >15 sec) and a floating baseline fHR that does not exceed 160 bpm.

#### Active state/HRP II (interpreted as active sleep 2F)

Fluctuating fHR with an oscillation bandwidth exceeding +/−5 bpm from floating baseline, frequent (at least 3 per 10 min) accelerations (>15 bpm, >15 sec), and the fHR exceeding 160 bpm only during accelerations.

#### Active state/HRP III (interpreted as active awakeness 4F)

fHR patterns with long-lasting accelerations exceeding 160 bpm, frequently fused into a sustained tachycardia (not analysed because of small sample size).

In the normal group, the classified and analysed data sets included n = 113 (63 females) in quiet (HRP I) and n = 286 (145 females) in active sleep state (HRP II). In addition, n = 29 data sets were classified HRP III (active awakeness). The resulting overall frequency distribution of observed fetal states by heart rate pattern [HRP I, HRP II, HRP III] was [0.27, 0.68, 0.05].

In the cohort of IUGR fetuses, the distribution was observed as follows: n = [4], [11,4] [HRP I, HRP II, HRP III] representing [0.21, 0.58, 0.21] of the related data sets. Owing to the small numbers, only those fetuses in active sleep (n = 11) were analysed.

The developing complex autonomic (neuro-vegetative) control was assessed by heart rate variability (HRV) characteristics according to universal developmental indices, namely fluctuation amplitude, complexity, and pattern formation (Table 2).

**Table 2:** Heart rate variability indices.

Parameter	Calculation	Interpretation
Increasing fluctuation amplitude		
amplitude	20–95 inter-quantile distance of detrended NN intervalseries	Fluctuation range of heart beat intervals above an approximated baseline
Increasing complexity		
gMSE(3)	Generalized Mutual Information at coarse graining level 3of NN interval series	Complexity of heart rate patterns essentially modulated by complex sympatho-vagal rhythms
Pattern formation		
skewness	Skewness of NN interval series	Asymmetry, contribution of vagal and sympathetic activity with their different time constants, decline of decelerations and formation of acceleration patterns
pNN5	Percentage of differences between adjacent NN intervalsthat are >5 ms.	Formation of vagal rhythms
VLF/LF	Ratio between very low (0.02–0.08 Hz) and low(0.08–0.2 Hz) Frequency band power	Baseline fluctuation in relation to sympatho-vagal modulations
Traditional fetal heart rate indices		
meanHR	Average over all NN interval related values of 60,000/NN	Mean heart rate in bpm

Fluctuation amplitude index based on interquantile distance was newly introduced in this work. It is robust against outlier and allowed more consistent results compared to those previously obtained by standard deviation of normal heart beat intervals (SDNN) [14].

Complexity reflecting complicated synergistic and antagonistic sympatho-vagal interactions was estimated using quantile based generalized mutual information at scale 3 of generalized multiscale entropy (gMSE) previously found appropriate [14].

Patterns of interest were

- The developing heart rate accelerations that are mainly attributed to sympathetic activation patterns are reflected in skewness [13], [14].
- Fast vagal rhythms that are reflected in the part of differences of successive heart beat intervals exceeding 5 ms (pNN5). This cutpoint value of the pNNx family was stronger age dependent than pNN10 and pNN20 found in a preparatory analysis of our fetal data set (not shown). Those low cutpoint values reflect the low fetal fluctuation range of heart beat intervals in comparison to pNN50 usually applied in adults HRV analysis [3].
- Baseline stability defined as ratio of very low frequencies fluctuations compared to predominant higher frequency rhythms (frequency bands according to [33]). A stable baseline can be understood as a kind of maturating “pattern” compared to the randomly fluctuating baseline in the premature fetus [14].

In addition to those universal developmental indices, we calculated the mean heart rate (meanHR) that represents an established traditional basal marker in clinical cardiotocography and fetal heart rate analysis.

### Statistical Models

The fetal age was predicted by multivariate linear regression models (forward procedure: stepwise inclusion of variables while P(F)<0.05; backward procedure: stepwise exclusion of variables while P(F)>0.1) for each state independently. The resulting models are considered as fetal autonomic brain age score (fABAS). The models were 70/30 split sample cross-validated. The state classification by experts is modeled by a cross-validated multivariate logistic regression model (weighted sample sizes). The contribution of factors was evaluated by ANOVA. P<0.05 was considered significant.

In a supplementary study, we explored whether IUGR fetuses can be distinguished from normal fetuses by means of fABAS as well as by mean heart rate (meanHR), respectively. Separate binary logistic regression models (weighted sample sizes) using [WGA, fABAS] and [WGA, meanHR] as predictors were fitted to estimate odds ratios (OR) with 95% confidence intervals (CI), Wald test statistic, its significance level, and rate of correct classifications.

## Results

### Normal Development

Univariate regression models were investigated for indices of the universal developmental aspects “increasing fluctuation amplitude”, “increasing complexity”, “pattern formation” as well as of the traditional heart rate index meanHR.

In the quiet sleep, heart rate traces all indices with exception of amplitude significantly predicted age in the univariate regression models. The strongest univariate predictors were gMSE3 and pNN5.

All parameters were considered in the stepwise multivariate modeling procedures. In the forward models only gMSE, skewness and VLF/LF were included as result. In the backward gMSE, skewness, VLF/LF, and pNN5 remained included, but with weak contribution of pNN5. The parameter gMSE strongest contributed to the prediction of developmental age followed by VLF/LF and skewness. The backward model explained 63/66% of the variance by age (Table 3). The parameters meanHR as well as amplitude did not contribute to the multivariate models.

**Table 3:** Univariate predictors and multivariate regression model in quiet state.

Parameter	R^2^	beta	Slope (95% CI)	p-value
**univariate**				
amplitude	0.03	0.16	0.29 (−0.04, 0.63)	0.087
gMSE3	0.54	0.73	39.90 (33.00, 46.85)	<0.001
skewness	0.20	0.44	2.06 (1.27, 2,85)	<0.001
pNN5	0.34	0.58	21.33 (15.73, 26.94)	<0.001
VLF/LF	0.23	−0.52	−1.15 (−1.50, −0.79)	<0.001
meanHR	0.16	−0.41	−0.22 (−0.31, −0.12)	<0.001
**Multivariate model**				
gMSE3	0.66/0.63	0.46	24.88 (16.72, 33.03)	<0.001
skewness		0.24	1.13 (0.56, 1.71)	<0.001
VLF/LF		−0.26	−0.56 (−0.84, −0.28)	<0.001
pNN5*		0.14	5.16 (−0.08, 10.39)	0.054

Coefficient of determination R^2^ (training set/validation set in the multivariate model), standardized regression coefficient beta, slope (95% confidence interval) of the models, significance.

* pNN5 was included only in the backward modeling.

In the active sleep, heart rate traces all indices with exception of VLF/LF significantly predicted age in the univariate regression models. The strongest univariate predictors were skewness and amplitude. Again, all parameters were considered in the stepwise multivariate modeling procedures. The forward and backward modeling results were identical. The parameters amplitude, skewness, and gMSE strongly contributed to the multivariate model, but also pNN5 and VLF/LF were considered according to the inclusion/exclusion criteria. In contrast, meanHR did not contribute to the multivariate models (Table 4).

**Table 4:** Univariate predictors and multivariate regression model in active state.

Parameter	R2	beta	Slope (95% CI)	p-value
**univariate**				
amplitude	0.34	0.57	0.41 (0.34, 0.48)	<0.001
gMSE3	0.05	0.23	12.84 (6.50, 19.18)	<0.001
skewness	0.35	0.59	4.01 (3.37, 4,65)	<0.001
pNN5	0.23	0.48	17.03 (135.36, 20.69)	<0.001
VLF/LF	0.00	0.02	0.03 (−0.19, 0.26)	0.766
meanHR	0.04	−0.21	−0.11 (−0.17, −0.05)	<0.001
**Multivariate model**				
amplitude	0.51/0.50	0.37	0.27 (0.18, 0.35)	<0.001
gMSE3		0.22	12.29 (5.70, 18.88)	<0.001
skewness		0.33	2.27 (1.59, 2.95)	<0.001
pNN5		0.11	3.76 (−0.59, 8.11)	0.090
VLF/LF		0.12	0.23 (0.05, 0.40)	0.012

Coefficient of determination R^2^ (training set/validation set in the multivariate model), standardized regression coefficient beta, slope (95% confidence interval) of the models, significance.

The multivariate models were considered fetal autonomic brain age score (fABAS).

**Figure 1:**
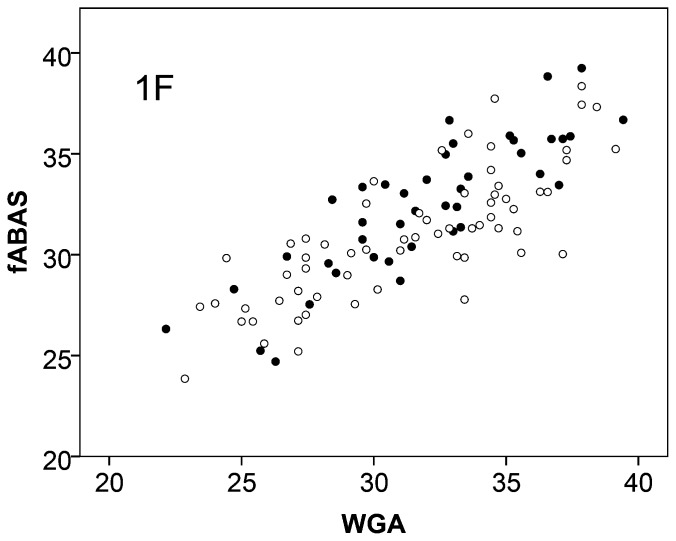
Fetal autonomic brain age score (fABAS) versus chronological age in quiet sleep of females ○ and males •. For gender comparison see Table 5.

**Figure 2:**
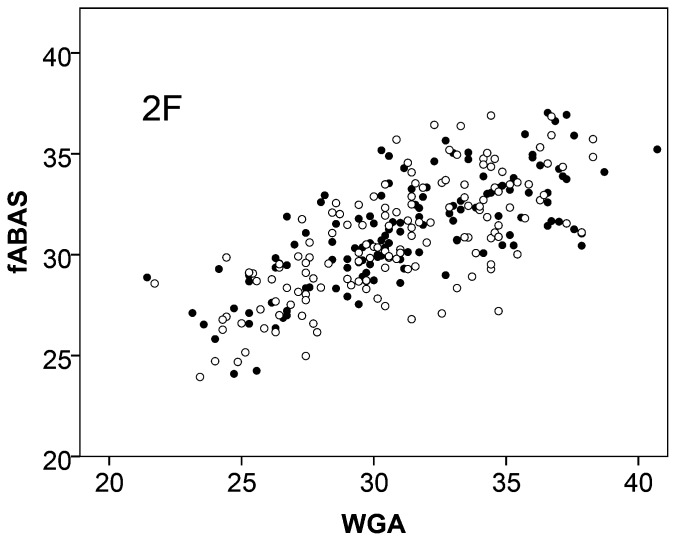
Fetal autonomic brain age score (fABAS) versus chronological age in active sleep of females ○ and males •. For gender comparison see Table 5.

With regard to gender dependencies, we found that fABAS increased slightly stronger in the male fetuses in the quiet sleep. The score was mainly (63.2%) explained by the fetal age (WGA) and to a small extend of 2.5% by the fetal sex. In contrast, in the active sleep the sex did not contribute at all (Figure 1, 2, Table 5).

**Table 5:** Contribution of WGA and sex to the variance of fABAS (ANOVA models).

source	Sum of squares	df	F	p-value
**Quiet:**				
WGA	736.34	1	197.47	<0.001
sex	29.31	1	2.96	0.006
total	1164.75	103		
**Active:**				
WGA	1073.1	1	289.02	<0.001
sex	4.17	1	1.123	0.290
total	2082.45	272		

For a future application of fABAS, an automatic state classification would be helpful. Due to the limited sample size available, only active versus quiet sleep was investigated here. The logistic regression models correctly reclassified an average of more than 90% of the states only by the amplitude index according to the expert decision. The inclusion of WGA in the model increased the correct classification rate only to a minor extend (Table 6).

**Table 6:** State classification results of logistic regression models.

	Training set			Validation set		
	quiet	active	Overall(%)	quiet	active	Overall(%)
amplitude	93.0	91.3	91.6	93.9	90.7	92.3
amplitude+WGA	94.7	94.1	94.4	93.7	92.4	93.1

### Changes Associated with IUGR

**Figure 3:**
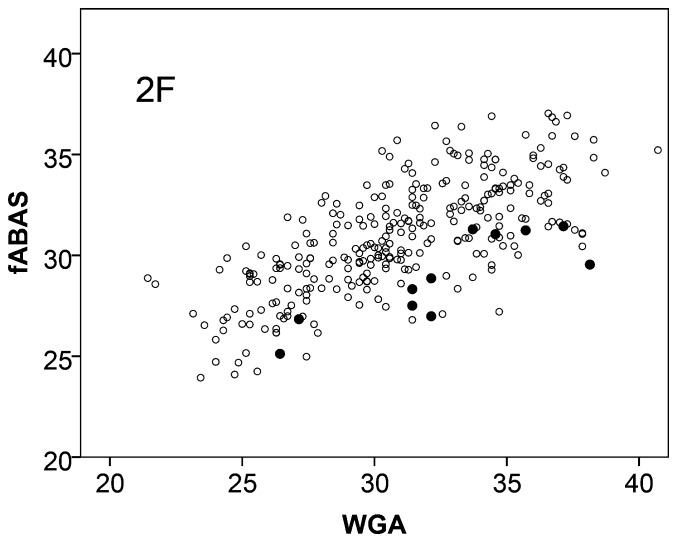
Fetal autonomic brain age score (fABAS) vs. chronological age in active sleep. Normals ○, intrauterine growth retarded (IUGR) group •. For group comparison see Table 7.

**Figure 4:**
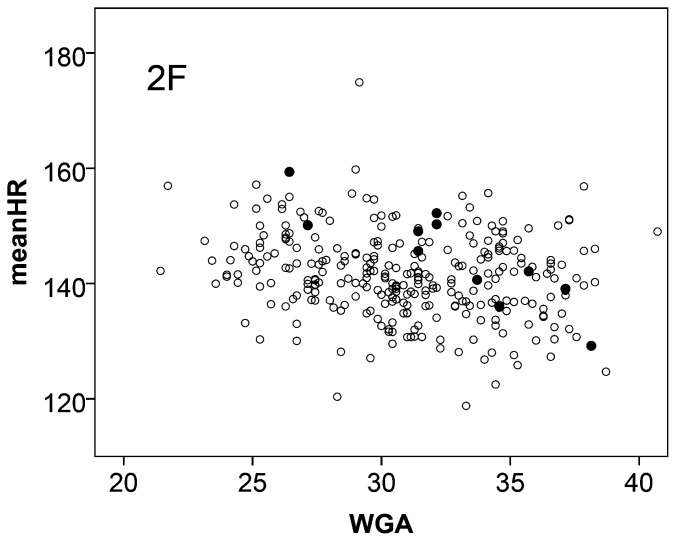
Mean heart rate (meanHR) vs. chronological age in active sleep. Normals ○, intrauterine growth retarded (IUGR) group •. For group comparison see Table 7.

IUGR influenced the fetal autonomic development in an asymmetric way. In comparison to normals the age score values were reduced while within the small sample studied here the sleep state distribution mimics a shift towards increased activity (HRP III). The value of fABAS to identify compromised fetal autonomic development was explored using 11 IUGR fetuses in active sleep (HRP II). IUGR was associated with clearly reduced fABAS values (Figure 3) and significant odds ratios of both, WGA and fABAS, in the bivariate logistic regression model (Table 7). Furthermore, the mean heart rate was increased, but less clear discriminative, as shown in Figure 4 and the bivariate logistic regression model [WGA, meanHR] (Table 7).

**Table 7:** Discrimination of IUGR and normal group by means of bivariate logistic regression models that include [fABAS, WGA] and [meanHR, WGA], respectively.

Measure	B	SE	Wald	p-value	OR (95% CI)	correct classifications
WGA	0.85	0.08	126	<0.001	2.34 (2.02, 2.72)	
fABAS	−1.37	0.12	135	<0.001	0.26 (0.20, 0.32)	
						82.3%
WGA	0.29	0.03	76	<0.001	1.34 (1.23, 1.43)	
meanHR	0.14	0.02	75	<0.001	1.15 (1.11, 1.18)	
						71.5%

Regression coefficient B, standard error SE, Wald test statistic with significance level p, odds ratio OR (95% confidence interval), correct classification rate.

## Discussion

The present work aimed at the assessment of fetal functional brain age with respect to ANS mediated control. Universal system developmental characteristics were found to contribute to a respective age score obtained from fetal heart rate patterns. Predominant characteristics are increasing fluctuation amplitude, increasing complexity and pattern formation.

- Fluctuation amplitude clearly increased in the active sleep data. The inter-quantile distance appeared as an advantageous fluctuation amplitude measure. The 20% percentile well approximated the baseline of heart rate fluctuations and the 95% percentile well approximated the heart rate acceleration amplitude. In the present data it predicted age better than SDNN. Nevertheless, the fetal age associated fluctuation amplitude raise is qualitatively consistent with other amplitude related indices previously reported in several studies, e.g. [12].
- The complexity at multiscale coarse graining level of 3 heart beat intervals is interpreted as result of complex sympatho-vagal modulations. The importance of that range of dynamics is consistent with results from multiscale sample entropy and Kullback Leibler (pattern entropy) functions of heart beat interval series as well as of equidistantly resampled heart rate data [14], [34]–[36]. It is remarkable that gMSE3 increased in both sleep states.
- Heart rate accelerations (AC) of increasing frequency, amplitude and duration are typical patterns that emerge and evolve in active sleep. Those asymmetric excitations were assessed by skewness. It is remarkable that also skewness increased in the quiet sleep. Other patterns are predominantly vagally mediated fast rhythms that are represented in pNN5. They increased in both states as well. According to the state definitions, a baseline stabilization expressed as declining VLV/LF could be shown in quiet sleep. All those pattern related results are consistent with previous univariate analyses from similar or related HRV characteristics e.g. [13], [14].

The novel multivariate models showed that those different aspects of universal system developmental characteristics contributed to state depending age score. Based on the selected developmental indices and the multivariate models, we developed the first scores of fetal developing autonomic control that explains a meaningful part of the variability by the fetal age.

Among others, Goldstein et al. [37] reported gender-dependent fetal programming of major depressive disorder and cardiovascular disease which occurred later in life. The small extent of slower development in females found in our quiet sleep data of normal fetuses without risk suggests rather no meaningful sex difference, but needs a validation from independent data. In a previous state-independent study no sex differences of fetal heart rate patterns were found [38].

The proposed methodology was appropriate, based on previous univariate results and the state of the art. In contrast to fetal magnetoencephalography (fMEG) recordings that provide direct access to the cortical brain function, but require an enormous high technical and methodological effort [39], fetal magnetocardiography (fMCG) recordings used here provide an indirect access to the activity of the ANS mainly controlled in the brain stem. Since recording longer than 30 min are hardly tolerable by pregnant women individual recordings include only one activity state or changing activity states. Therefore, both selected states were separately investigated and an intra-individual combination was not done. The established semi-quantitative way of state classification by experts seems to be partly improvable by logistic regression models which consider a tendency of smaller fluctuation amplitudes in the younger age. An extension to all possible fetal states seems possible. Here, recording periods without a clear stationary state over at least 10 min in HRP I or HRP II were not selected. The univariate linear regression models of all investigated HRV indices over the entire investigated maturation period could be improved by nonlinear (quadratic) models by less than 10% only in the present data (not shown here). Therefore, we did not use nonlinear characteristic curves in the present multivariate models. Our present work was focused at the system theoretic developmental indices. Nevertheless, a subdivision into maturation segments before and after WGA 31, as proposed by [40], may be able to improve those models. The HRV indices were selected from the variety of more or less correlated indices published in the last decade and the youngest own results. Similar results may be obtainable from other related indices. As far as we know we propose the first multivariate cross-validated age score of the fetal developing autonomic control. The remaining residuals are in the range of expected physiological variability of behavior.

So far, the age dependence of HRV indices (univariate analysis with exception of the composite indices of sympatho-vagal balance SDNN/RMSSD, VLF/LF, VLF/HF, LF/HF, see [24], [33] based on magnetocardiographic recordings were reported to range from partly only trendlike relationships up to significant ones (e.g. [13], [40]. The quantitative heterogeneity of the reported results may mainly be attributed to the differences in investigated data length, the consideration of fetal behavioural states, and the handling of artifacts and non-stationarities. Furthermore, the small sample sizes may not have allowed sufficient cross validation in those explorative studies. The multivariate age score, introduced herein, improved the age dependency compared to the univariate models presented here as well as the published state of the art including composite indices of sympathovagal balance. Furthermore, the calculation of the indices amplitude, skewness, gMSE3, and pNN5 using histogram and quantile characteristics, respectively, makes them robust against artifacts and outliers. This is a potential advantage in analyzing measured data with all their flaws. Finally, it should be taken into consideration that heart rate patterns reflect not only fetal age but also a variability of individual fetal behavioural patterns at the particular age. The investigated 30 min recordings are snapshots in that regard. A significantly longer magnetocardiographic recording time is hardly tolerable for a pregnant woman, this restricts the assessment of all possible behavioural states further.

In contrast to the developmental indices described here, related parameters obtained by computerized cardiotocography (cCTG), namely basal fetal heart rate, short-term and long-term heart rate variations, analyzed in a cross sectional study of 4412 fetuses [41] did not permit developmental age assessment. The limitations of the established cCTG are twofold: (i) the restricted temporal resolution in Doppler based cardiotocography while fetal magnetocardiography and electrocardiography deliver a sampling precision of 1 ms, and (ii) the lack of considering system theoretically motivated universal developmental indices like introduced in the present work.

The present results support our hypothesis that interpretation of HRV according to the concept of universal system developmental characteristics can help to understand and quantitatively evaluate fetal brain development. Universal system developmental principles explain the formation of structure in any dissipative system, such as shown in primitive living matter, coupled neurons, up to the evolution of highly complex organisms. Those principles provide a link between general evolution and individual genetically predetermined and individually adapted ontogeny. Within the genetically determined range intrauterine influences to the fetus can modulate the individual development by means of epigenetic re-programming. It is likely that similar developmental principles also apply in that context. The present findings seem to support this approach. Appropriate consideration of those principles may not only facilitate early identification of fetal developmental disturbances. Furthermore, it may have implications for designing innovative prophylactic and therapeutic strategies.

The explorative results of IUGR-related alterations of autonomic development indicate changes concerning fetal behavioural state distribution and HRV characteristics. The small sample size limited the possibilities for HRV analysis to HRP II only. Both, the shift towards appearance of HRP III (active awakeness) and the increased heart rate in HRP II (active sleep) can be explained by increased sympathetic activation associated with IUGR. The lower WGA dependent fABAS values found in HRP II may reflect alterations in the emerging complex autonomic control system in terms of the considered universal developmental aspects “increasing fluctuation amplitude”, “increasing complexity”, and “pattern formation”. A systematic investigation of those universal developmental indices under inclusion of all fetal behavioural states in more detail may be able to further generalize the system theoretic approach introduced here. Respective extension and validation of the present results, which require a clearly larger sample size, is subject of our further work.

## Conclusions

In summary, the functional maturation of the ANS mediated control in the fetus was successfully assessed by system theoretically motivated indices of universal principles of system development applied to heart rate patterns. Based on multivariate models for active and quiet sleep of normally developing fetuses, the “fetal autonomic brain age score” (fABAS) was proposed. The general validity of the investigated universal developmental indices provides a novel view on fetal autonomic brain development.

This approach may have implications for early identification and more comprehensive understanding of fetal developmental disorders as well as for designing novel concepts in prophylaxis and therapy.
